# The Asclepiad

**Published:** 1884-03

**Authors:** 


*The Asclepiad.* A quarterly Journal. Vol. I, No I., pp. 96. By BENJAMIN WARD RICHARDSON, M.D., F.R.S. CADE AND CAULFIELD. London: January 1884.

Under this high sounding title Dr. Richardson, as editor and sole contributor, presents us with the first number of a new quarterly. We are not quite sure whether the name of "asclepiad" is intended to suggest a relation with the priestly followers of the god of medicine himself or is only intended to uphold the doctrines of the well-known Roman physician Asclepiades, who was such a successful preacher of the little-flesh-no-wine-much-exercise rule of living. If we might draw conclusions from the nature of the contents of the work before us we should suppose the latter. Rome in the days of Asclepiades was in many respects similar to London in the days of Richardson. Let us hope that the modern teacher may be as successful as the old one was.

Dr. Richardson's writings in this journal are of a piece with his more recent productions generally, and belong to that doubtful borderland between grave medicine and light literature which a worried practitioner finds most comfortable reading in the arm-chair after dinner. It is easily understood, moderately true, somewhat novel, and not overcrowded with statistics and facts. We feel when we have laid it down that if we have learnt little we have whiled away a pleasant hour in a legitimate manner, free from the torment of lethal germs and the travails of German pathology.

We like Dr. Richardson's journal. The author is one of the few men in our profession who can write the language of a cultured Englishman, and this alone ought to commend his work to our readers. "Felicity as a sanitary research" is the subject of one of his articles, and if his efforts do not add to our happiness they do not—as the efforts of most of our scribbling confreres do—add to our miseries; and this is a good deal to say in favour of any part of the medical language and literature of the present day. We wish the venture every success, and hope that Dr. Richardson may live to give us many such volumes.

